# Divergent contributions of multiple PPIases to cell survival, sociality, and stress tolerance in *Myxococcus xanthus* DK1622

**DOI:** 10.1128/aem.01252-26

**Published:** 2026-08-31

**Authors:** Tian-yu Wan, Rui-yun Chen, Zi-ye Zhou, Ying Cao, Zhuo Pan, Yue-zhong Li, Li Zhuo

**Affiliations:** 1 State Key Laboratory of Microbial Technology, Institute of Microbial Technology, Shandong University 214177, Qingdao, China; Michigan State University, East Lansing, Michigan, USA

**Keywords:** peptidylprolyl isomerase, social behavior, stress tolerance, functional divergence, *Myxococcus*

## Abstract

Peptidylprolyl isomerase (PPIase) catalyzes the rate-limiting step of proline *cis-trans* isomerization during protein folding. In bacteria, multiple PPIases are commonly present and participate in diverse physiological processes. *Myxococcus xanthus* DK1622 possesses as many as 17 PPIase genes. We previously reported the functional divergence of the four trigger factor family PPIases. Here, we systematically assessed the contributions of the remaining PPIases in cell survival, sociality, and stress tolerance. Pin1, localized in the periplasm, was essential for cell growth. Absence of the membrane-bound Pin3 impaired the social motility, predation ability, and sporulation but did not affect the fruiting body formation of *M. xanthus*. Furthermore, nearly all *M. xanthus* PPIases contributed to tolerance to environmental stresses. The transcriptional levels and expression patterns of these PPIases varied distinctly, even among members of the same family or with high sequence homology. Our findings provide a comprehensive view of functional divergences and suggest a potential functional cooperation network of PPIases in *M. xanthus*.

**IMPORTANCE:**

Peptidylprolyl isomerase (PPIase), a ubiquitous enzyme present across nearly all kingdoms of life, catalyzes the *cis-trans* isomerization of peptidyl-prolyl bonds in polypeptide chains, thereby significantly accelerating protein folding. As a result, PPIases play critical roles in a wide range of physiological processes mediated by their substrate proteins. Myxobacteria are distinguished by their complex multicellular social behaviors. Among the 17 PPIases belonging to three families in *Myxococcus xanthus* DK1622, the membrane-bound Pin3, one of the SurA homologs, is involved in social behaviors such as social motility, predation, and sporulation. Given that PPIases are widely recognized as chaperones, our results also indicate that these *M. xanthus* PPIases extensively contribute to stress tolerance. Our findings underscore the essential functions of PPIases in cellular processes and reveal a correlation between the expansion of cellular functionalities and the functional evolution of PPIase proteins.

## INTRODUCTION

Correct folding of proteins into three-dimensional structures is essential for their functional competence, and two types of proteins play critical roles in assisting this complex process. One is the molecular chaperone system, which initiates assistance in protein folding as the nascent polypeptide chain emerges from the ribosome, thereby protecting partially folded intermediates and preventing protein aggregation (1). The other type of enzymes, known as “folding catalysts,” enable the pushing of the rate-limiting steps of protein folding, such as peptidylprolyl isomerases (PPIases) (2, 3). PPIases can catalyze the isomerization of proline-peptide bonds (Xaa-Pro) in oligopeptides, greatly accelerating the protein folding process (4, 5).

PPIases are a group of proteins that possess a conserved peptidylprolyl isomerase domain (PPD) and are ubiquitously distributed across all cellular organisms (6). According to the affinity of PPD to different drugs, PPIases are classified into three structurally distinct superfamilies: FK506-binding proteins (FKBPs), cyclophilins (Cyps), and parvulins (Pins) (7). With the exception of a few PPIase members that consist solely of the core PPD domain, most PPIases are supplemented by N-terminal or/and C-terminal extensions, which confer additional functional capabilities (6). For example, the ribosome-associated molecular chaperone trigger factor (Tig), which is a component of the molecular chaperone system belonging to FKBP-type PPIase, possesses the ribosome binding domain at the N-terminal and the substrate binding domain at the C-terminal (8). PPIases are ATP-independent enzymes (9, 10) that are located in the cytoplasm, periplasm, or in the membrane of gram-negative bacteria. In *Escherichia coli*, the parvulin family SurA, the FKBP family FkpA, and the cyclophilin family PpiA are located in the periplasmic space (10–12). Additionally, the parvulin family PpiD has been identified at the cell membrane (13).

PPIases play important roles in various cellular processes, including transcription, protein transport, cytotoxicity, and stress response (10, 14, 15). Many PPIases have been confirmed to possess the chaperone functions in protein refolding and prevention of aggregation (12, 16, 17). In the periplasmic space of *E. coli*, SurA and FkpA are critical for the structural stability and translocation of outer membrane proteins (OMPs) and secretory proteins (12, 17). Moreover, PPIases are closely associated with bacterial survival and virulence (15, 18–23), underscoring their potential as targets for antibacterial development.

Myxobacteria are a group of gram-negative bacteria renowned for their distinctive social behaviors and abundant natural products (24–26). In contrast to the predominantly unicellular life cycle exhibited by most bacteria, myxobacteria display unique social motility and “wolf-pack” group predation. Under nutrient-limiting conditions, myxobacterial cells undergo aggregation and orchestrate the formation of multicellular fruiting bodies, wherein a subset of cells differentiate into stress-resistant myxospores (12, 27). The model strain *Myxococcus xanthus* DK1622 possesses a large genome and harbors an extensive number of duplicate genes (28). We previously characterized the multiple genes of the molecular chaperone system and elucidated their intrafamilial functional divergence in *M. xanthus* DK1622 (29–32). In contrast to 10 PPIases found in the gram-negative *E. coli* and 4 PPIases in the gram-positive *Bacillus subtilis*, a total of 17 PPIases belonging to the three major families were identified in *M. xanthus* DK1622. In this study, we investigated the contributions of this unusually large set of PPIases to the complex cellular behavior and environmental stress tolerance of *M. xanthus*, with the aim of elucidating the reasons for their genomic retention. We have previously reported cellular functions and evolutionary patterns of the four Tig family proteins; these proteins belong to the FKBP-type PPIases (31). To investigate the functions of the remaining 13 PPIases, we individually knocked out the encoding genes of these PPIases in *M. xanthus* DK1622, and detected the cell phenotypes of these deletion mutants, particularly the social behaviors and stress responses. Our results revealed distinct phenotypic defects among the deletion mutants, indicating that these PPIases are involved in diverse physiological processes of *M. xanthus* DK1622. These findings contribute to the understanding of the functional divergence and potential cooperative mechanisms of multiple PPIases in bacteria.

## RESULTS

### *Myxococcus xanthus* DK1622 possesses 17 PPIases

**Fig 1:**
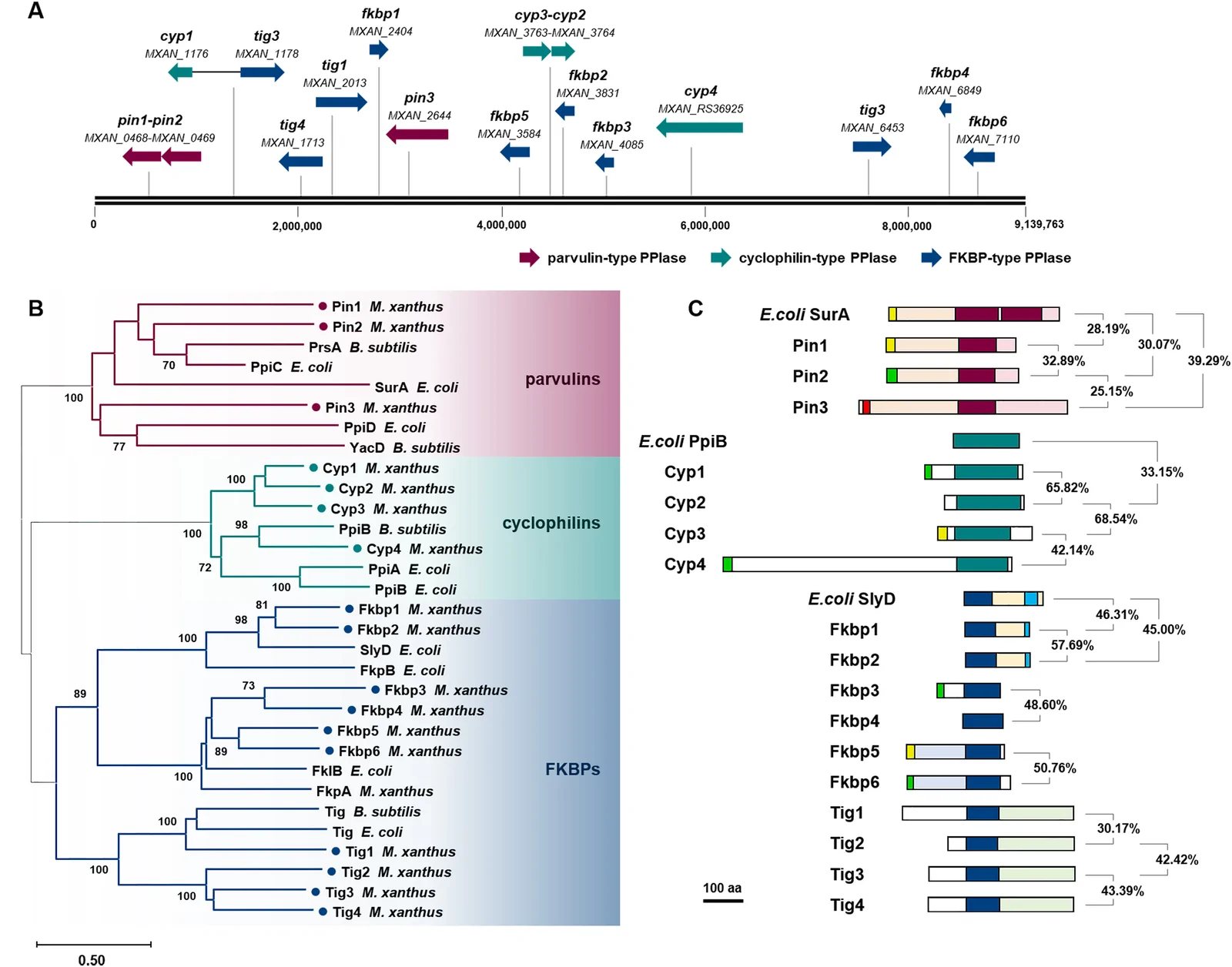
Bioinformatic analysis of PPIases in *M. xanthus* DK1622. (**A**) Genomic locations of the 17 PPIase-encoding genes in *M. xanthus* DK1622. (**B**) Phylogenetic analysis of PPIases in *M. xanthus* DK1622, *E. coli*, and *B. subtilis*. Support values larger than 70% are presented. (**C**) Protein domain diagram of these PPIases. Sequence identity percentages for closely related proteins are provided. Sec/SPI-type signal peptides are highlighted in yellow; Sec/SPII-type signal peptides are in green; transmembrane domains are in red; and poly-His regions are in blue. The dark regions are the PPI domains, and the shared light-colored regions in N- or C-terminal extensions indicate similar sequences.

According to the NCBI annotation and conservative domain analysis, 17 PPIase-encoding genes were retrieved in the genome of *M. xanthus* DK1622 (Fig. 1A; Table S1). In terms of *E. coli* and *B. subtilis* PPIases for references, phylogenetic analysis of these PPIases indicated that 17 *M. xanthus* PPIases were clearly clustered into three branches, with each branch precisely corresponding to parvulin, cyclophilin, and FKBP families, respectively (Fig. 1B). Thus, the three parvulin-type PPIases were designated as Pin1-3; the four cyclophilin-type PPIases were termed Cyp1-4; and the remaining six FKBP-type PPIases (excluding the four Tigs reported before [31]) were named Fkbp1-6. These 17 *M. xanthus* PPIases presented certain associations in the genetic distribution (Fig. 1A), suggesting their functional and transcriptional relationship and synergy. For example, *pin2* and *pin1* exhibit adjacent distribution in the same orientation, a pattern that is also observed between *cyp3* and *cyp2*. In contrast, *cyp1* and *tig3* are oriented in opposite directions, with an uncharacterized functional gene located between the two loci. The amino acid sequence analysis revealed that the PPDs among these 17 PPIases were relatively conserved within each family (Fig. S1), whereas significant sequence differences were observed in the extensions at the N-terminal or C-terminal (Fig. 1C; Fig. S2). Unlike the periplasmic PPIase SurA of *E. coli*, containing double PPDs, all three *M. xanthus* parvulin PPIases had a single PPD, and their sequence identities were approximately 30% to each other (Fig. S1). Fkbp1 and Fkbp2 of *M. xanthus* DK1622, which were phylogenetically closely related to *E. coli* SlyD, exhibited a sequence identity of 57.69% and possessed incomplete poly-His tails at the C-terminal (sequence shown in Fig. S2).

Bioinformatics analysis revealed that only Pin3 had a transmembrane domain, and except for the four Tigs and Cyp2, all the other PPIases with N-terminal extensions had a signal peptide at the N-terminal (Fig. 1C; Table S2). *E. coli* PpiD also possessed the transmembrane domain, which was exactly the most similar to Pin3. Cyp2, without a signal peptide at the N-terminal, was consistent with *E. coli* PpiB, a main contributor to PPIase activity within the cell. A Sec/SPI signal peptide was predicted at the N-terminal of Pin1, Cyp3, and Fkbp5, whereas Pin2, Cyp1, Cyp4, Fkbp3, and Fkbp6 might contain an N-terminal Sec/SPII signal peptide. Different localizations provide some cues to cellular functionalities of PPIases to some extent (33).

The PPDs of *M. xanthus* PPIases possessed distinct protein isoelectric points (pI), despite their relatively conserved sequences (Table S2). Even when the pI values of PPDs were similar, those of the complete PPIases differed due to the presence of extensions. For example, the pI values of whole Pin1 and Pin2 were similar; the PPD of Pin1 was acidic, whereas that of Pin2 was basic. Variations in pI among homologous proteins may affect substrate affinity, potentially driving their functional divergence. These findings suggested that different characteristics of the N-terminal and C-terminal extensions may contribute to differences in cellular localization and functional specificity among PPIases, even within the same family.

### The periplasmic Pin1 is essential for strain survival

**Fig 2:**
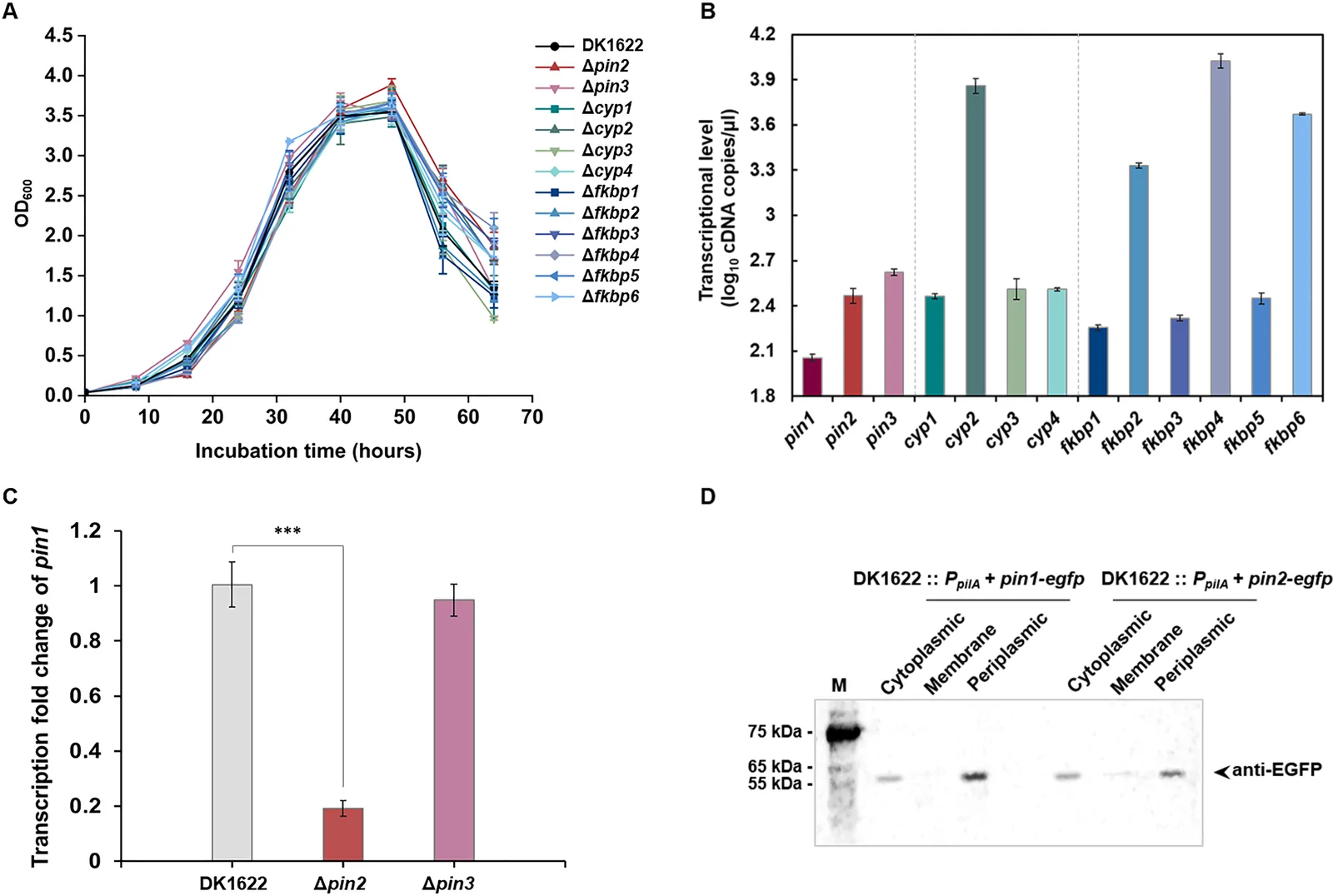
Transcription and cellular localization of the indispensable PPIase Pin1. (**A**) Growth curves of PPIase-encoding gene deletion mutants under normal conditions. (**B**) Transcriptional analysis of PPIase-encoding genes at mid-log growth phase (24 h incubation). The *Y*-axis shows the log_10_ value of the transcription quantity. The Ct*_gapA_* value of each sample was set as 25 for sample normalization. (**C**) Relative transcriptional fold changes of *pin1* in Δ*pin2* and Δ*pin3* mutants at 24 h compared to wild-type strain DK1622, which was set as reference level 1. (**D**) Western blot analysis detecting the presence of the target protein in different cellular fractions of the recombinant mutants.

We attempted to individually delete the coding genes of these PPIases in *M. xanthus* DK1622. Despite multiple experimental efforts, the *pin1* deletion mutant could not be generated, whereas all the other deletion mutants were successfully constructed, obtaining a total of 16 single-gene knockout strains of PPIases, including the four Tig deletion mutants constructed before (31). We found that the knockout of these deletable PPIase-encoding genes did not affect the normal growth of the strain (Fig. 2A). To verify the essentiality of *pin1* in *M. xanthus* DK1622, we used the pSWU30 plasmid to insert an additional copy of the *pin1* gene at the *attB* site of *M. xanthus* DK1622, followed by the knockout of the original *pin1* gene. The comparable growth ability of this knockout strain to that of the wild-type DK1622 (Fig. S3) verified that the protein function of Pin1 was essential for the survival of *M. xanthus* DK1622. Nevertheless, the transcriptional level of *pin1* at mid-log phase was the lowest among all PPIase-encoding genes (Fig. 2B), even lower than the average level of the entire transcriptome in the strain (34). *pin1* and *pin2* were located adjacently on the genome (Fig. 1A); we found that deletion of *pin2* significantly reduced the transcriptional level of *pin1* compared to the wild-type strain, whereas the *pin1* expression remained nearly unchanged in the Δ*pin3* mutant (Fig. 2C). However, the deletion of *pin2* had no obvious effect on the growth ability of the mutant (Fig. S3). These results suggested that the necessity of Pin1 for cell growth was determined by its function, rather than by its transcriptional abundance.

The sequence analysis above indicated that, within the parvulin family, Pin1 and Pin2 both possessed signal peptide sequences, while Pin3 had a transmembrane region at the N-terminal. We sought to isolate the periplasmic, membrane, and intracellular protein fractions of *M. xanthus* DK1622 (Fig. S4), and identified the presence of PPIases in each fraction by mass spectrometry (MS) analysis. Consistent with the prediction of an N-terminal Sec/SPI signal peptide, Sec/SPII signal peptide, or transmembrane domain, Pin1 was detected in the periplasmic fraction; Pin2 was detected in the cell membrane fraction; and Pin3 was also detected with substantial abundance in the cell membrane fraction (Data Set S1). We subsequently constructed the *M. xanthus* mutants to express recombinant proteins of Pin1 and Pin2 fused with EGFP at the C-terminal. Further Western blot results showed that both Pin1 and Pin2 proteins were present in the periplasmic fraction (Fig. 2D). Pin2 was also detected in the crude membrane fraction, a distribution pattern that is likely attributable to its Sec/SPII signal peptide. These findings confirm that Pin1 and Pin2 are capable of being secreted into the periplasmic space. Unlike parvulin-type PPIase in *E. coli*, only PpiC existed in the cytoplasm (35); there was no parvulin-type PPIase located in the cytoplasm in DK1622. Despite the fact that the transcriptional level of *pin1* was relatively low, the inability to delete this gene suggested its crucial function in the periplasm.

### Absence of *pin3* impairs the social behavior of *M. xanthus*

**Fig 3:**
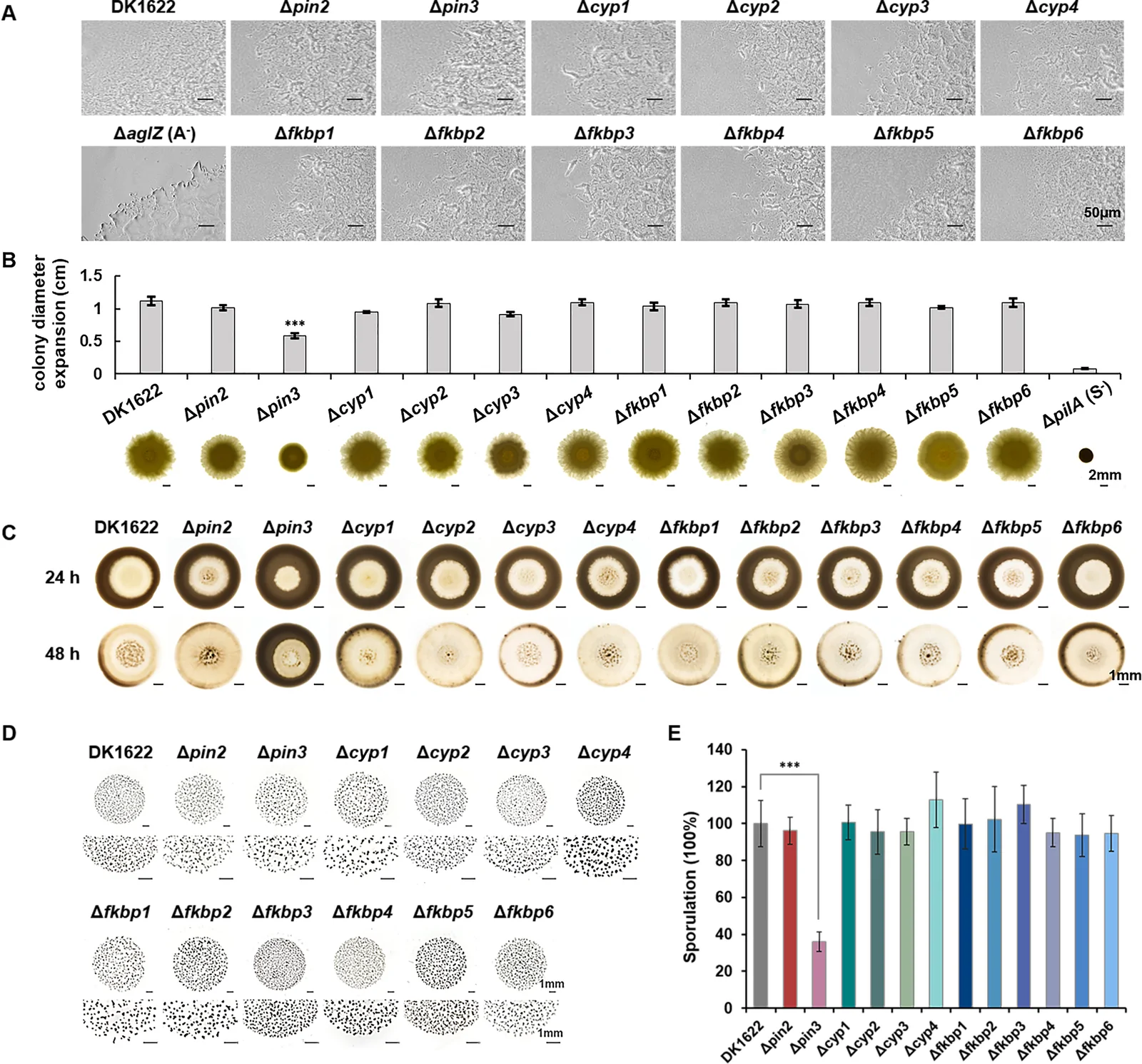
Social behavior analysis of PPIase deletion mutants in *M. xanthus* DK1622. (**A**) A-motility of deletion mutants. The formation of isolated colonies distant from the swarm edge was observed with a phase contrast microscope. The Δ*aglZ* strain (A-S+) served as a negative control. Scale bars: 50 μm. (**B**) S-motility of deletion mutants. Expansion of the swarm edge on 0.4% agar CTT plates was observed with a stereoscopic microscope at 48 h. The expansion distance of colonies was measured (*n* = 3). The Δ*pilA* strain (A^+^S^−^) was used as a negative control. Scale bars: 2 mm. (**C**) Predation activity of deletion mutants on *E. coli* prey mats. The predation zones were observed with a stereoscopic microscope at 24 and 48 h. Scale bars: 2 mm. (**D**) Fruiting body formation of these deletion mutants. Scale bars: 1 mm. (**E**) Sporulation efficiency of the deletion mutants relative to the wild-type strain DK1622. For statistical analysis, ***, *t*-test, *P* < 0.001.

Myxobacteria possess a characteristic social behavior and a complex life cycle, and the unique motility mechanism serves as the foundation for its multicellular behavior (27). On solid agar plates, *M. xanthus* cells rely on two distinct motility systems to coordinate group movement: social motility (S-motility), characterized by the spread of colonies, and adventurous motility (A-motility), shown as the outward expansion of individual bacterial cells (36). We used A-motility defective Δ*aglZ* and S-motility defective Δ*pilA* strains as references along with the wild-type DK1622 to evaluate the motility of the PPIase knockout mutants. Microscopic examination revealed that, on 1.5% agar plates, individual cells extended outward from the colony edges of each PPIase mutant (Fig. 3A), similar to that in the Tig family PPIase knockout strains (31), which indicated that single deletion of the PPIase gene in *M. xanthus* DK1622 did not significantly affect A-motility. However, the colony expansion of Δ*pin3* on 0.4% agar plates was significantly reduced compared to that of the wild-type strain (Fig. 3B), and complementation with an extra copy of *pin3* in the deletion mutant restored S-motility (Fig. S5). Nevertheless, the Δ*pin3* mutant exhibited a certain degree of colony expansion compared to the Δ*pilA* strain, which completely lacked S-motility. This result suggested that the absence of Pin3 impaired, but did not abolish, the S-motility of *M. xanthus* cells.

As a predatory bacterium, myxobacteria employ a wolf-pack group strategy to hunt bacterial prey (37). We used *E. coli* as the prey cells, which were smeared on nutrient-free TPM plates, according to the previous study (38). The results demonstrated that the deletion of *pin3* also led to an obviously smaller predation zone, while the other mutant strains exhibited a similar predation phenotype as the wild-type strain over the same incubation period (Fig. 3C). *M. xanthus* predation on bacterial prey on solid medium depends on its cell-group movement (37, 39). Therefore, the decreased predation rate of the Δ*pin3* mutant toward *E. coli* prey seems to be associated with its impaired S-motility.

Under nutrient-limited conditions, *M. xanthus* cells can aggregate and develop into multicellular fruiting bodies, with some cells differentiating into spherical, stress-resistant dormant spores (40). Nutrient-free TPM plates were used to induce sporulation of *M. xanthus* cells under starvation conditions. We observed that all the deletion mutants could develop fruiting bodies, but several mutants showed sparse fruiting body density (Fig. 3D). After 5 days of starvation induction, fruiting bodies were harvested and subjected to heat treatment to inactivate vegetative cells, followed by enumeration of viable spore colonies. The results indicated that only the *pin3* knockout strain exhibited a significantly reduced sporulation rate (Fig. 3E), which is similar to the absence of Tig3 or Tig4 of the FKBP family PPIase (31).

### Multiple PPIases are involved in the stress tolerance of *M. xanthus*

**Fig 4:**
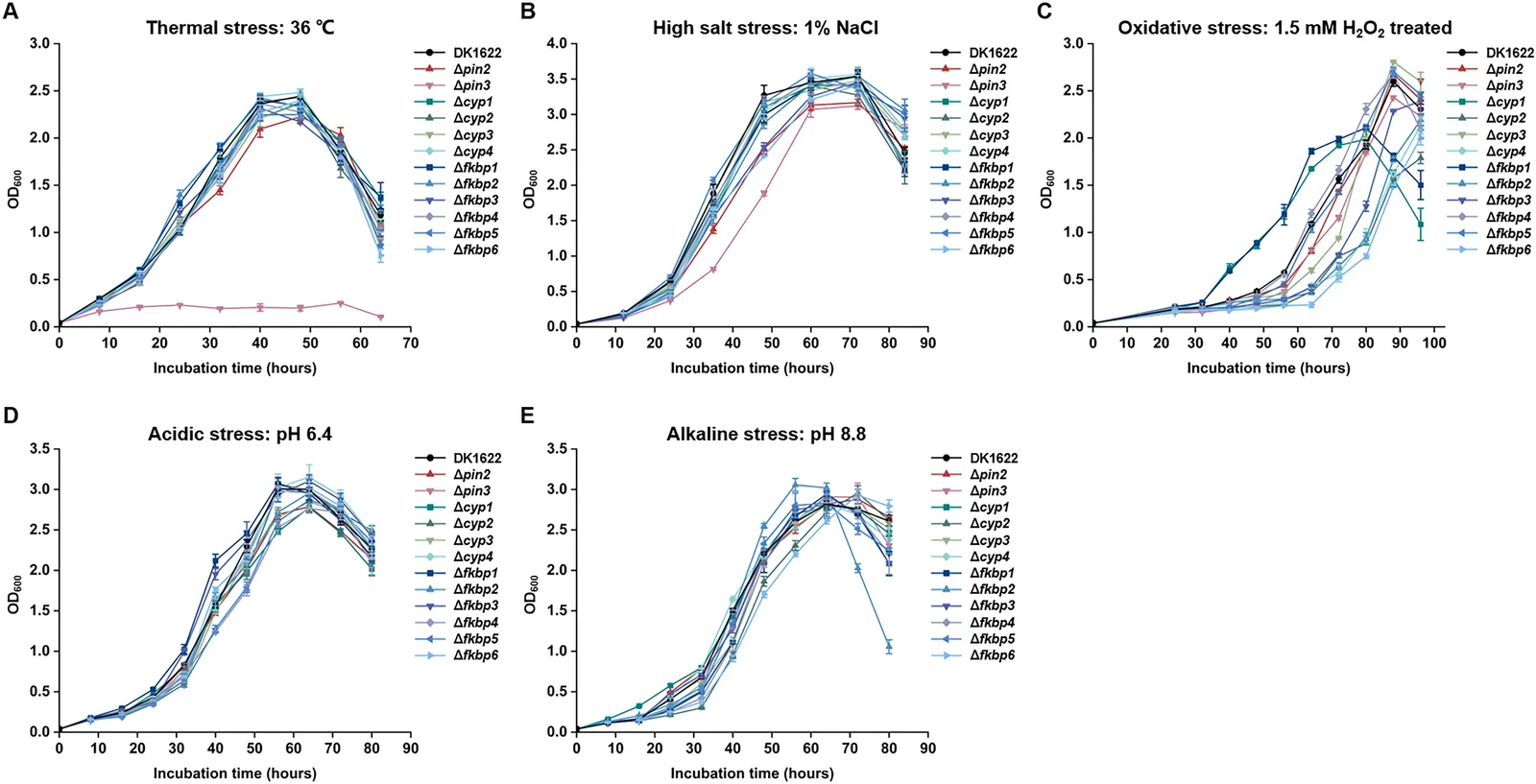
Growth analysis of PPIase deletion mutants under various stress conditions. (**A**) Growth curves of the PPIase deletion mutants under thermal stress at 36°C. (**B**) Growth curves of the PPIase deletion mutants under high salt conditions with 1% NaCl supplementation. For statistical analysis, the *P* values of the *t-test* were 0.0004 for Δ*pin2*, 0.0001 for Δ*fkbp3*, and 0.00003 for Δ*fkbp6* at 48 h compared to the wild-type DK1622, respectively. (**C**) Growth curves of the mutants following treatment with 1.5 mM hydrogen peroxide (H_2_O_2_) for 30 min. Growth curves of the mutants under (**D**) acidic conditions when the culture medium was adjusted to pH 6.4 and (**E**) alkaline conditions when the culture medium was adjusted to pH 8.8. Under pH 6.4 conditions, the *P* values of the *t*-test were 0.014 for Δ*fkbp4* at 40 h, 0.012 for Δ*fkbp4* at 48 h, 0.006 for Δ*fkbp5* at 40 h, and 0.016 for Δ*fkbp5* at 48 h compared to DK1622, respectively. Under pH 8.8 conditions, the *P* values of the *t*-test were 0.0496 for Δ*cyp2* and 0.0013 for Δ*fkbp6* at 40 h compared to DK1622.

The cellular functions of PPIases are commonly associated with the stress response and tolerance in microbial strains (14). Accordingly, we investigated the growth ability of each PPIase deletion mutant of *M. xanthus* under various stress conditions. Although the growth of these mutants under normal conditions was consistent with that of the wild-type DK1622 (Fig. 2A), the Δ*pin3* mutant exhibited severely impaired growth at an elevated temperature of 36°C (Fig. 4A), indicating that *pin3* was essential for thermotolerance. In addition, the absence of *pin3* also led to growth inhibition under high-salt conditions, whereas deletion of *pin2*, *fkbp3*, or *fkbp6* exerted only mild effects (Fig. 4B; *t*-test, *P* value <0.001 at 48 h).

Following the hydrogen peroxide treatment, the growth status of the two parvulin-type PPIase deletion mutants, Δ*pin2* and Δ*pin3*, was similar to that of the wild-type DK1622. In contrast, deletion mutants of the cyclophilin family, *cyp2*, *cyp3*, or *cyp4*, exhibited growth retardation. Similarly, deletion of the FKBP family *fkbp2*, *fkbp3*, or *fkbp6* also resulted in delayed growth after H_2_O_2_ treatment (Fig. 4C). The above cyclophilin-type and FKBP-type PPIases played an important role in the oxidative stress response of *M. xanthus* cells. Notably, similar to the observations in Δ*tig3* and Δ*tig4* (31), the Δ*cyp1* and Δ*fkbp1* mutants exhibited more rapid growth recovery than the wild-type strain following oxidative stress; however, their maximum cell density remained lower than that of the wild-type strain.

The Δ*fkbp4* and Δ*fkbp5* mutants exhibited growth inhibition under acidic culture conditions (Fig. 4D; *t*-test, *P* value <0.05 at 40 and 48 h), whereas the Δ*cyp2* and Δ*fkbp6* mutants displayed impaired growth in an alkaline environment (Fig. 4E; *t*-test, *P* value <0.05 at 40 h). We further constructed ectopic complementation mutants derived from the corresponding gene deletion mutants, and most of them exhibited recovery from the phenotypic defects caused by the original gene deletion (Fig. S6). Additionally, the FKBP family mutants Δ*tig1*, Δ*tig3*, and Δ*tig4* demonstrated growth inhibition at high temperature, while Δ*tig2* showed significant growth retardation after oxidative treatment (31). The above results indicated that, with the exception of *fkbp1* and *cyp1*, whose deletion did not result in any observable phenotypic defect under the tested stress conditions, all other PPIase genes were involved in the tolerance to specific environmental stresses of *M. xanthus*.

### Transcriptional analysis of multiple PPIases in *M. xanthus*

**Fig 5:**
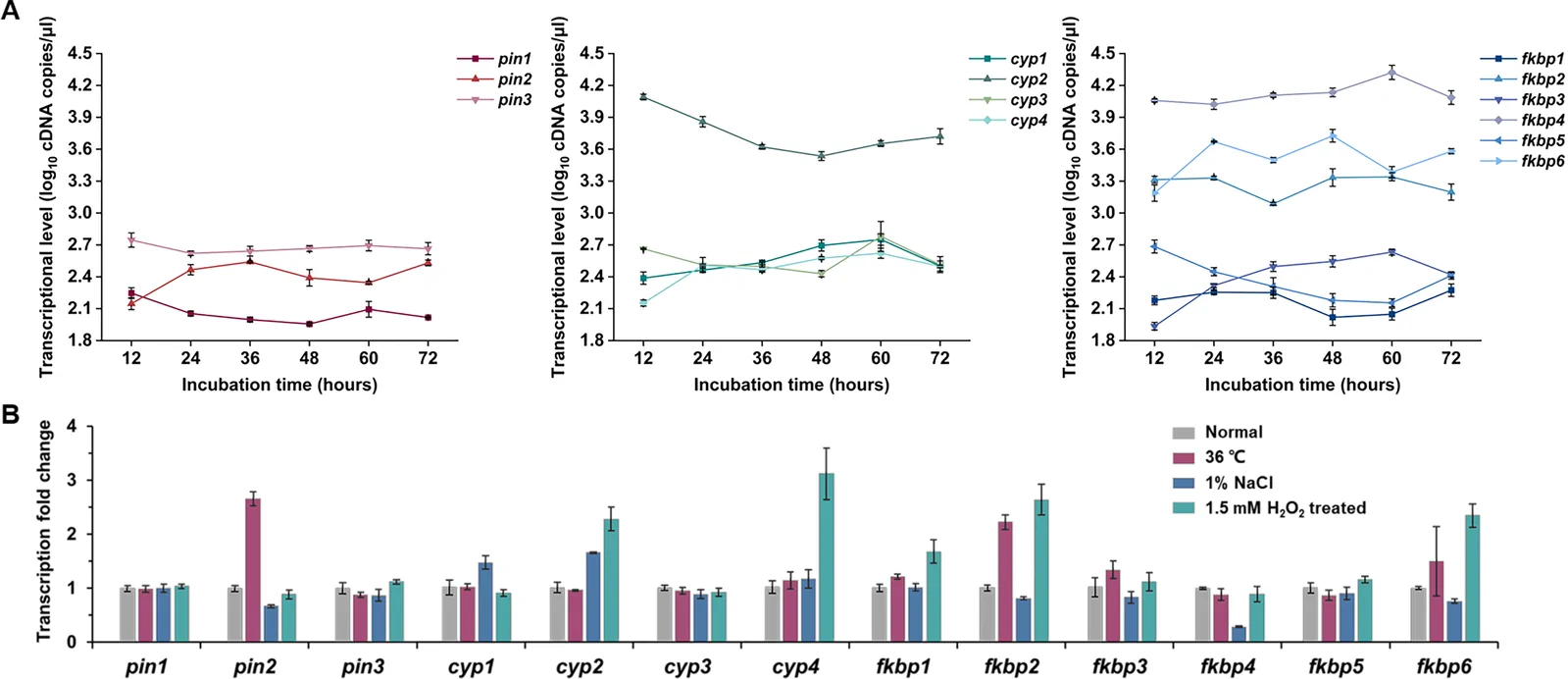
Transcriptional analysis of PPIase-encoding genes in *M. xanthus* DK1622. (**A**) Temporal changes in the transcriptional levels of these genes in the wild-type strain during incubation. (**B**) Relative fold changes in the expression of PPIase-encoding genes in *M. xanthus* DK1622 under stress conditions. The transcriptional level of each gene under normal conditions was normalized to a value of 1, and *gapA* was used as the reference gene.

We performed quantitative analysis of the transcriptional levels of each PPIase-encoding gene in *M. xanthus* DK1622 at different growth stages, i.e., 12, 24, 36, 48, 60, and 72 h after incubation, corresponding to the logarithmic growth, stationary, and decline phases of culture in CTT medium (32). Transcriptional analysis showed that both *fkbp4* and *cyp2* were highly expressed (Fig. 5A), which was consistent with the previous transcriptome data (34). The transcriptional level of *fkbp4* remained consistently high over the entire period, while *cyp2* exhibited significant fluctuations across different growth stages. In *E. coli*, the single-domain PPIase PpiB has been identified as the primary contributor to peptidylprolyl isomerase activity in the cytoplasm (35). *M. xanthus* Fkbp4 and Cyp2 also possess the PPD domain without extension domains, the high expression of their encoding genes suggesting that they may primarily serve to provide peptidylprolyl isomerase activity within the cell.

*cyp3* and *cyp2* were located adjacently with a 3 bp intergenic region (Fig. 1A), but the latter *cyp2* exhibited a 14-fold higher transcriptional level than *cyp3* at 24 h of incubation time. The absence of *cyp3* did not significantly affect the transcription level of *cyp2* (Fig. S7), suggesting that the variation in transcription levels might be attributed to differential mRNA degradation rates or intrinsic regulatory elements within the *cyp3-cyp2* locus. *fkbp6* and *fkbp2* were moderately expressed, while the remaining PPIase genes were generally expressed at low levels, especially *pin1*, the essential gene for cell growth, displaying particularly minimal transcription. The *fkbp1* gene was also expressed at an extremely low level, and no phenotypic defects were detected in the Δ*fkbp1* mutant, probably attributable to the functional redundancy among FKBP-PPIases. However, because the overall compensatory effect of these PPIase genes at the transcriptional level was not obvious under normal conditions (Fig. S7), the lowly expressed PPIases might function in some unknown specific biological processes.

Transcriptional analysis at different incubation time points revealed that *fkbp5* and *cyp2* exhibited similar expression trends, characterized by continuous downregulation during the early stage, followed by upregulation at the later stage. In contrast, only *fkbp3* exhibited a significantly increased transcription, with an approximate fivefold rise from 12 to 60 h, and displayed a downregulated trend during the decline phase. The adjacent genes *pin1* and *pin2*, both harboring an N-terminal signal peptide, exhibited opposite transcriptional trends, suggesting that their expressions were strictly regulated to ensure optimal function in the periplasm. The transcriptional levels and expression patterns of these PPIases indicated that the cellular requirements for these genes in *M. xanthus* varied across different growth stages.

We further investigated the transcriptional changes of these PPIase genes in *M. xanthus* DK1622 under different stress conditions by calculating the fold change relative to their expression levels under the normal condition (Fig. 5B). The transcriptional levels of *pin2* and *fkbp2* were upregulated under high-temperature conditions, although the knockout of these two genes did not affect the strain growth at elevated temperature. These results suggest that both genes might be involved in the thermal stress response of *M. xanthus*, even though their functions could be compensated by other genes in the corresponding deletion mutants. *cyp2*, *fkbp2*, and *fkbp6* were upregulated following oxidative treatment, which was consistent with the delayed growth phenomenon after oxidative treatment occurring in the corresponding knockout strains. These results indicated that multiple *M. xanthus* PPIases were involved in the stress response at the transcriptional level.

## DISCUSSION

PPIases are widely recognized as chaperone proteins that assist in the folding, stabilization, transport, and translocation of proteins, as well as playing roles in microbial stress tolerance (14). Myxobacteria display more complex cellular processes than other bacteria, as evidenced by their cooperative behaviors, including group predation and the formation of multicellular fruiting bodies, which are processes linked to their distinctive motility patterns (41). Consistent with this complexity, myxobacteria typically possess large genomes and numerous duplicate genes. Previously, we have characterized the functional redundancy and divergence of multiple genes in different molecular chaperone families (29–31). Here, we extend our analysis to examine the cellular functions of the 13 uncharacterized PPIases in *M. xanthus* DK1622. Together with the four PPIases belonging to the Tig family (31), these 17 PPIases are broadly implicated in social behaviors and stress tolerance of *M. xanthus*, revealing extensive functional redundancy alongside notable functional divergence among them.

The conserved PPD of PPIase exhibits intrinsic peptidylprolyl isomerase activity, while N-terminal or C-terminal extensions endow these proteins with additional chaperone activities and specialized biological roles (6). For instance, the periplasmic chaperone SurA plays important roles in protein folding and quality control (9). In *Bacteroides fragilis*, a periplasmic PPIase homologous to *E. coli* SurA has been identified as an essential gene required for mediating bacterial intraspecies competition (42). Among the three parvulin family PPIases in *M. xanthus* DK1622, Pin1 and Pin2 were located in the periplasm, whereas Pin3 was membrane associated. Unlike SurA in *E. coli* or *B. fragilis* that contains two PPDs, PrsA and YacD, the homologs of SurA in gram-positive *B. subtilis*, both have only one PPD (43, 44). PrsA, the more conserved homolog than YacD, is essential for the growth of *B. subtilis* (43). All three parvulin family PPIases of *M. xanthus* DK1622 also possess only a single PPD like *B. subtilis*. Importantly, Pin1 is essential for normal growth, while Pin3 is required for growth under high-temperature stress and contributes to social behaviors in *M. xanthus*, suggesting clear functional divergence among these three paralogs.

In *E. coli*, Tig, FklB, PpiD, and SurA all participate in the synthesis and translocation of secreted proteins, whereas Tig, FkpA, PpiA, PpiB, PpiD, and SurA are associated with the biogenesis and structural integrity of the outer membrane (6, 15, 35, 45). In the periplasm, the defect in biofilm formation caused by PpiA deficiency can be rescued by complementation with either FkpA or PpiD (10). Furthermore, FkpA and SurA exhibit functional redundancy in the biosynthesis of OMPs under heat shock conditions (17). Under thermal stress, the transcription of *pin2* in *M. xanthus*, a periplasmic SurA homolog encoding gene, was induced by heat shock; however, the growth of *pin2* deletion mutant remained comparable to that of the wild-type DK1622. In contrast, the deletion of the other SurA homolog, Pin3, led to a severe impairment in growth at elevated temperatures. These findings suggest that the cellular function of Pin2 can be effectively compensated under heat stress, whereas the function of Pin3 cannot, underscoring its unique contribution to thermotolerance. Similarly, multiple PPIases in *M. xanthus* DK1622, including the four Tig family members, are broadly implicated in the tolerance of the strain to diverse stresses such as oxidative, acidic, alkaline, and saline conditions.

**Fig 6:**
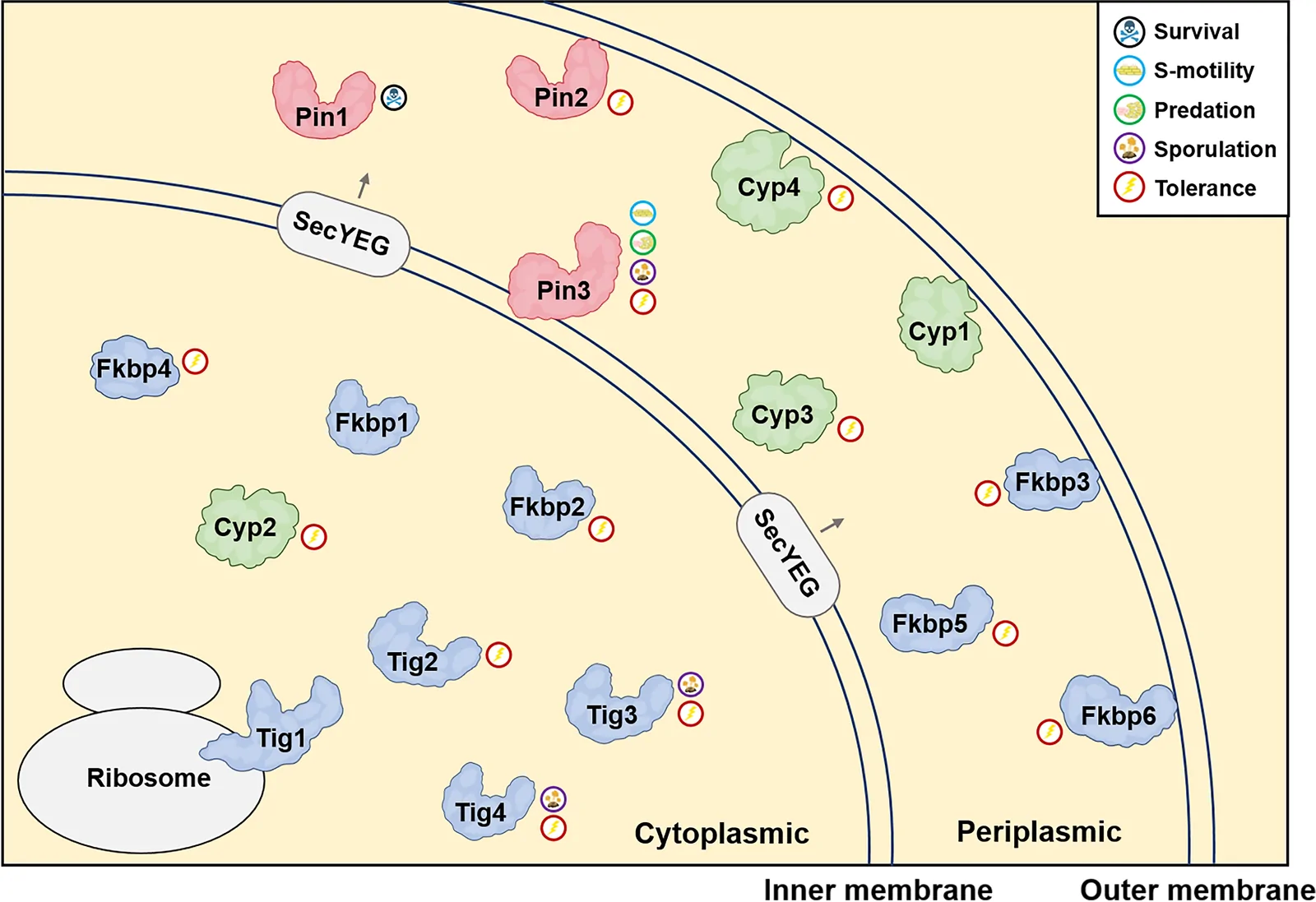
Schematic diagram of the putative localization of PPIases in *M. xanthus* DK1622 and the phenotype of the corresponding deletion mutants. The localization was inferred from protein sequence analysis and cellular fractionation data.

The properties of the extended domain often determine the localization patterns and substrate engagement of PPIases (6). Among the four Tig homologs in *M. xanthus* DK1622, only Tig1 is capable of ribosome binding and acts as the ribosome-associated molecular chaperone (31). The other three copies lack the specific N-terminal motif required for ribosome binding and thus are no longer considered ribosome-associated molecular chaperones. In particular, when signal peptides or transmembrane regions are present within these extensions, they may dictate the specific subcellular localization and functional specialization of PPIases, allowing them to engage in cellular pathways like protein secretion. Based on sequence analysis and MS data of cell fractionation (Data Set S1), the putative localization of multiple PPIases in *M. xanthus* DK1622 has been determined. As shown in Fig. 6, with the exception of the four PPIases belonging to the Tig family, almost all PPIases containing an N-terminal extension region are predicted to localize to the periplasmic space or the cellular membrane. This finding further underscores the extensive involvement of PPIases in protein biogenesis, secretion, and homeostasis within the periplasmic compartment. This phenomenon may be attributed to the ATP-independent nature of PPIases, rendering them particularly advantageous in energy-limited environments of the periplasm in gram-negative bacteria. Except for Fkbp1 and ribosome-associated Tig1, almost all other PPIases exhibited certain contributions under stress conditions. In particular, only Pin3, the sole membrane-bound SurA homolog in *M. xanthus* DK1622, has been associated with the distinctive social behaviors and stress tolerance of *Myxococcus*. Thus, PPIases play an important role in the environmental adaptability of *M. xanthus*. It is reasonable to hypothesize that *M. xanthus* DK1622 has retained 17 PPIases to coordinate its complex cellular processes. Despite the limited scope of the association analysis conducted in this study, the findings from single-deletion mutants highlight differential involvement in cellular processes and unequivocally establish the existence of functional divergence across homologous PPIase proteins. Further research is required to elucidate their specific functional distinctions and potential cooperative mechanisms.

## MATERIALS AND METHODS

### Strains, plasmids, and growth conditions

The strains and plasmids used in this study are listed in Table S3. The *M. xanthus* strains were cultivated in casitone-based rich-nutrient (CTT) medium (1% casitone, 8 mM MgSO_4_, 10 mM Tris-HCl [pH 7.6], and 1 mM K_2_HPO_4_-KH_2_PO_4_ [pH 7.6]) (46). TPM medium (10 mM Tris-HCl [pH 7.6], 8 mM MgSO_4_, 1 mM K_2_HPO_4_-KH_2_PO_4_, and 1.5% agar) (47) was used as the starvation condition for *M. xanthus* strains. The *E. coli* strains were grown in Luria-Bertani medium (tryptone 1%, yeast extract 0.5%, and NaCl 1%) routinely. Agar (1.5%) was supplemented for the solid medium. *M. xanthus* and *E. coli* were cultured at 30°C and 37°C, respectively, unless otherwise noted. If selection was necessary, kanamycin at a final concentration of 40 μg/mL or tetracycline (Tet) at a final concentration of 10 μg/mL was added to the medium.

### Bioinformatics analysis of PPIase in *M. xanthus* DK1622

According to NCBI (https://www.ncbi.nlm.nih.gov/) genome annotation and protein sequences, there are 17 PPIases in *M. xanthus* DK1622. Phylogenetic trees were constructed based on protein sequences of these 17 proteins and the PPIases in *E. coli* and *B. subtilis* by using MEGA 11. Sequence alignment was produced by using the online program MAFFT (https://www.ebi.ac.uk/Tools/msa/mafft/) and embellished by ESPript (https://espript.ibcp.fr/ESPript/cgi-bin/ESPript.cgi). Sequence identity was analyzed by NCBI Protein BLAST (https://blast.ncbi.nlm.nih.gov/Blast.cgi). The Expasy database (https://www.expasy.org/) was used to calculate the isoelectric point and molecular weight of the protein, as well as to predict transmembrane regions. Signal peptides were predicted by SignalP 6.0 (https://services.healthtech.dtu.dk/services/SignalP-6.0/).

### Construction of plasmids and strains

The primers used for PCR amplification are listed in Table S4.

Gene deletion in *M. xanthus* DK1622 was performed by using positive-negative KG cassettes as previously described (48). The deletion plasmids were constructed by cloning 800 bp upstream and downstream homologous fragments of the gene into pBJ113. The deletion plasmid was transformed into *M. xanthus* DK1622 cells by electroporation, and colonies with Km resistance were first selected. Then, 1.5% D-galactose (Sigma) was used for the second selection. Finally, the deletion mutants were confirmed by PCR and sequencing.

The *pin1* complementation mutant of *M. xanthus* was constructed by combining the gene after the *pilA* promoter into the *attB* site-specific recombination plasmid pSWU30 for *Myxococcus*. A single colony with Tet resistance was selected and evaluated.

The remaining PPIase complementation mutants were generated by heterotopically inserting an additional copy of the corresponding gene downstream of the *pilA* promoter into the *attB* site of the *Myxococcus* genome using the plasmid pSWU19.

### Extraction and fractionation of *M. xanthus* cellular proteins

The extraction and fractionation of *M. xanthus* cells refer to the method in *Yersinia pseudotuberculosis* and *Acinetobacter baumannii*, with some modifications (49, 50).

*M. xanthus* strains were cultured in liquid for 24 h to the mid-logarithmic phase. Cells were collected by centrifugation and washed three times. Cells were resuspended in Tris-HCl buffer (25 mM Tris-HCl and 200 mM NaCl [pH 7.6]) containing 20% sucrose and incubated with gentle shaking at 4°C for 30 min. The cells were centrifuged and then resuspended in Tris-HCl buffer and incubated with gentle shaking at 4°C for 1 h. The cells were centrifuged again at 8,000 rpm for 30 min at 4°C to obtain the periplasmic protein fraction in the supernatant. The cell sedimentation was resuspended and broken by ultrasonication, then centrifuged at 10,000 rpm to collect the supernatant. The cells were ultracentrifuged at 4°C at 45,000 × *g* for 1 h, and the supernatant components and the sedimentation components were collected separately to obtain the cytoplasmic fractions and the crude membrane fractions.

Proteins were identified by MS. The cytoplasmic protein trigger factor, membrane-bound PilQ, and cytoplasmic DegP were selected as markers for detecting the separation results. MS data indicated that the periplasmic fraction was contaminated by cytoplasmic components, possibly due to cell lysis during its isolation.

### Western blot analysis

The western blot analysis was performed using standard procedures as described previously (51). We fused the EGFP gene at the C-terminus of the *pin1* or *pin2* genes into the autonomous replication plasmid pZJY4111 to construct *M. xanthus* recombinant strains that express the fusion protein with an EGFP tag. After obtaining proteins in different cell spaces of these recombinant strains, samples were separated on SDS-PAGE. After that, proteins were transferred to PVDF membranes. The target proteins were detected by anti-EGFP.

### Growth curves under different conditions

The *M. xanthus* strains were cultured in liquid CTT medium at 30°C and 200 rpm for approximately 20 h to the logarithmic phase as seed cells. The cells were then transferred to 50 mL of CTT liquid medium with a final concentration of OD_600_ = 0.04 and cultured at 30°C and 200 rpm, and a small part of the culture medium was removed every 8 h to measure the OD_600_.

The stress conditions tested in this study were chosen according to a previous study (29). A temperature of 36°C was employed as the thermal pressure condition. For the oxidative stress assay, given that H_2_O_2_ is prone to decomposition in the culture medium, the seed culture concentration was adjusted uniformly to 3 × 10^8^ cells/mL (OD_600_ = 1). Subsequently, the cells were treated with H_2_O_2_ at a final concentration of 1.5 mM for 30 min. The treated cells were then inoculated into a normal medium to investigate the involvement of these PPIases in the oxidative stress response and the post-oxidation recovery of *M. xanthus* strains. For the high-salt environment, 1% NaCl was added to the CTT liquid. The pH = 6.4 and pH = 8.8 of CTT liquid were chose as acidic and alkaline environment pressure.

### *M. xanthus* social behavior detection

The social behaviors of *M. xanthus* that we detected here include A- and S-motility, predation, and fruiting body development. Assays were performed according to previous methods (29, 30, 36).

The *M. xanthus* strains were cultured to the logarithmic phase and centrifuged to collect the cells. The cells were washed three times with TPM buffer (10 mM Tris-HCl, 8 mM MgSO_4_, and 1 mM K_2_HPO_4_-KH_2_PO_4_ [pH 7.6]) and adjusted to a final concentration of 1 × 10^10^ cells/mL (adjust to OD_600_ = 1 and then 35 times concentrated). Droplets (5 μL) were cultured on 1.5% CTT solid plates for A-motility detection, and 5 μL droplets were cultured on 0.4% CTT solid plates for S-motility detection.

The concentration of *E. coli* was adjusted to 1 × 10^11^ cells/mL (adjust to OD_600_ = 1 and then 100 times concentrated) by TPM. *E. coli* cells (35 μL) were dropped onto a TPM plate to form a colony approximately 1 cm in diameter. Then, 5 μL of condensed *M. xanthus* was dropped into the center of the *E. coli* colony. The rate of *M. xanthus* expansion was used to characterize the predation ability of *M. xanthus* on *E. coli*.

To detect the fruiting body formation and myxospore production of *M. xanthus*, 8 μL of condensed *M. xanthus* cells was dropped onto a non-nutritive TPM plate and cultured at 30°C for 5 days. The fruiting bodies were observed by stereoscopic microscopy. Subsequently, the fruiting bodies were dispersed in 1 mL of TPM and heated at 50°C for 2 h to kill the vegetative cells. After ultrasonic treatment, the spore suspension was diluted on CTT plates and cultured at 30°C. The rates of sporulation were calculated by the number of colonies growing on the CTT plates.

### Quantitative PCR analysis

Quantitative PCR analysis was carried out as described previously (29). Cellular RNA was obtained with a bacterial total RNA extraction kit (Thermo Fisher Scientific, USA). Then, reverse transcription was carried out with HiScript Q-RT SuperMix for qPCR (Vazyme) after genome removal. The cDNA was subsequently subjected to qPCR analysis using AceQTM Universal SYBR qPCR Master Mix (Vazyme). *gapA* (a glyceraldehyde-3-phosphate dehydrogenase-encoding gene, *MXAN_2815*) was used as the reference. Here, we measured the linear curve of the concentration versus cycle threshold (Ct) values of each pair of primers and set Ct*_gapA_* = 25 for normalization of each sample.

The quantitative PCR primers are listed in Table S4.

## Data Availability

The experimental data sets generated in this study are provided in the article and supplemental material or are available from the corresponding author upon reasonable request for non-commercial scientific purposes, including verification, reproduction, or reanalysis. The genome and associated sequence data of *Myxococcus xanthus* DK1622 are publicly available in the NCBI GenBank under accession no. CP000113.1 (RefSeq accession no. NC_008095.1).

## SUPPLEMENTAL MATERIAL

Data Set S1 aem.01252-26-s0001.xlsx MS-based identification of proteins in different fractions of *M. xanthus* DK1622.

Supplemental material aem.01252-26-s0002.pdf Figures S1 to S6, Tables S1 to S4, and legend for Data Set S1.

ASM does not own the copyrights to Supplemental Material that may be linked to, or accessed through, an article. The authors have granted ASM a non-exclusive, world-wide license to publish the Supplemental Material files. Please contact the corresponding author directly for reuse.
