# Supplementary material for: Divergent contributions of multiple PPIases to cell survival, sociality, and stress tolerance in Myxococcus xanthus DK1622
Source: Appl Environ Microbiol. 2026 Aug 31;92(9):e01252-26. doi: 10.1128/aem.01252-26 (PMC13599543; doi:10.1128/aem.01252-26)
Supplement: Supplementary file 1 [file aem.01252-26-s0002.pdf]

Supplementary Information for

Divergent contributions of multiple PPIases to cell survival, sociality, and stress tolerance in *Myxococcus xanthus* DK1622

Tian-yu Wan[$], Rui-yun Chen[$], Zi-ye Zhou, Ying Cao, Zhuo Pan, Yue-zhong Li*, Li Zhuo*

[$] These authors contribute equally

* The corresponding authors

Emails: zhuoli1992@sdu.edu.cn, lilab@sdu.edu.cn

This PDF file includes:

Figures S1 to S7

Tables S1 to S4

Legend of Data S1

Supplementary Figures

A

Coverage / Identity	Pin1	Pin2	Pin3
Pin1		83% / 91%	42% / 51%
Pin2	32.89%		46% / 52%
Pin3	37.41%	31.28%	

B

Coverage / Identity	Cyp1	Cyp2	Cyp3	Cyp4
Cyp1		90% / 79%	78% / 76%	19% / 63%
Cyp2	65.82%		75% / 90%	18% / 76%
Cyp3	75%	68.54%		17% / 60%
Cyp4	42.02%	38%	42.14%	

C

Coverage / Identity	Fkbp1	Fkbp2	Fkbp3	Fkbp4	Fkbp5	Fkbp6
Fkbp1		93% / 96%	47% / 47%	58% / 38%	31% / 48%	
Fkbp2	57.69%					
Fkbp3	25%	-		99% / 67%	48% / 71%	41% / 69%
Fkbp4	27.42%	-	48.6%		42% / 98%	40% / 98%
Fkbp5	27.85%	-	35.83%	46.3%		75% / 76%
Fkbp6	-	-	37.27%	47.62%	50.25%	

D

Coverage / Identity	TF1	TF2	TF3	TF4
TF1		55% / 42%	64% / 53%	41% / 35%
TF2	30.17%		82% / 96%	85% / 98%
TF3	27.12%	42.24%		96% / 95%
TF4	27.15%	39.42%	43.39%	

Figure S1. Homology analysis of protein sequences among PPIases within the same family. The blue-shaded numbers indicate the identity percentage of the homologous regions. The green- and red-shaded numbers represent the proportion of sequence length corresponding to the homologous regions of the proteins labeled in matching colors.

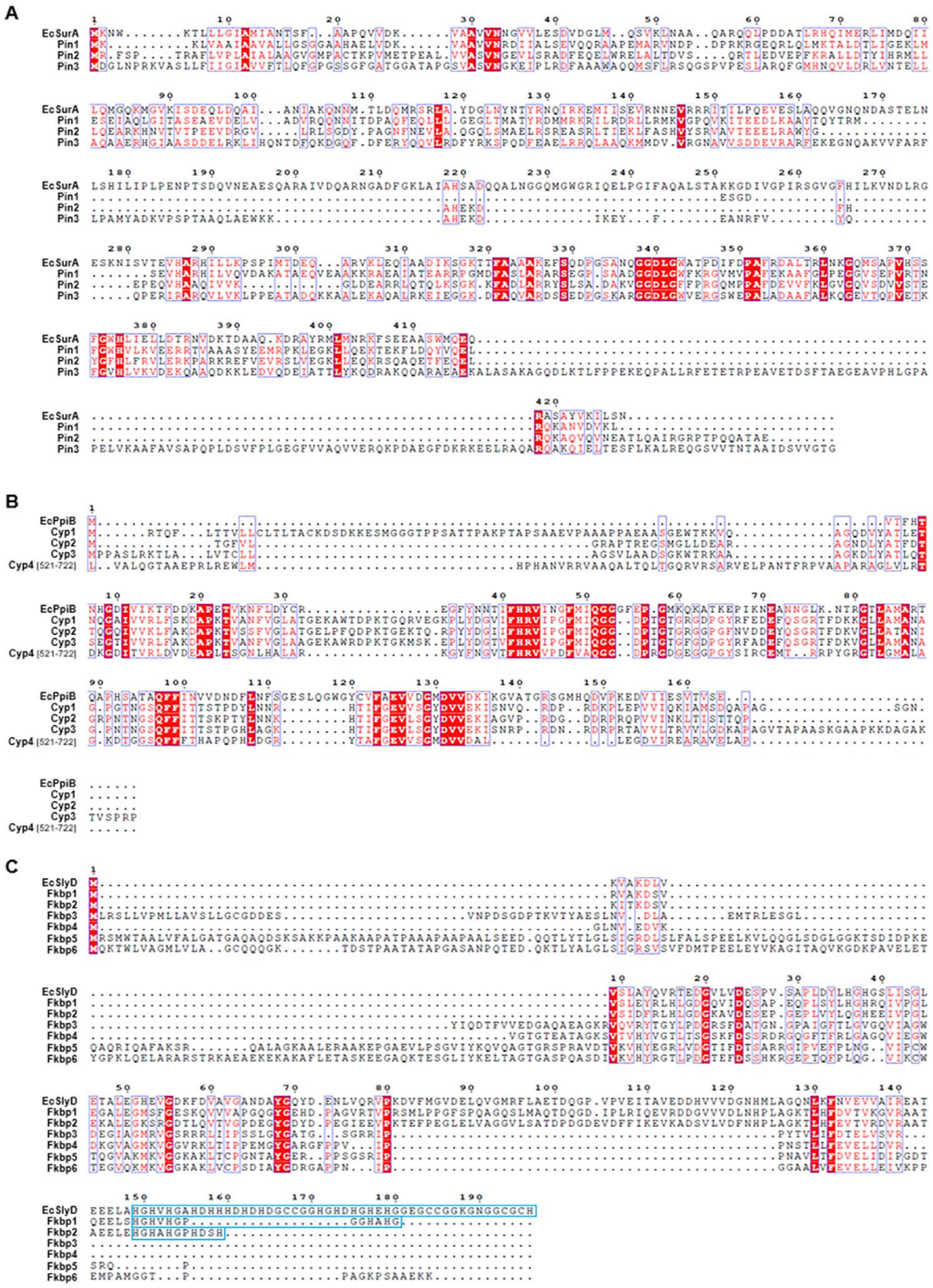

Figure S2. Sequence alignment of PPIases in *M. xanthus* DK1622. Parvulin-type SurA, cyclophilin-type PpiB, and FKBP-type SlyD from *E. coli* were used as reference sequences for their corresponding families.

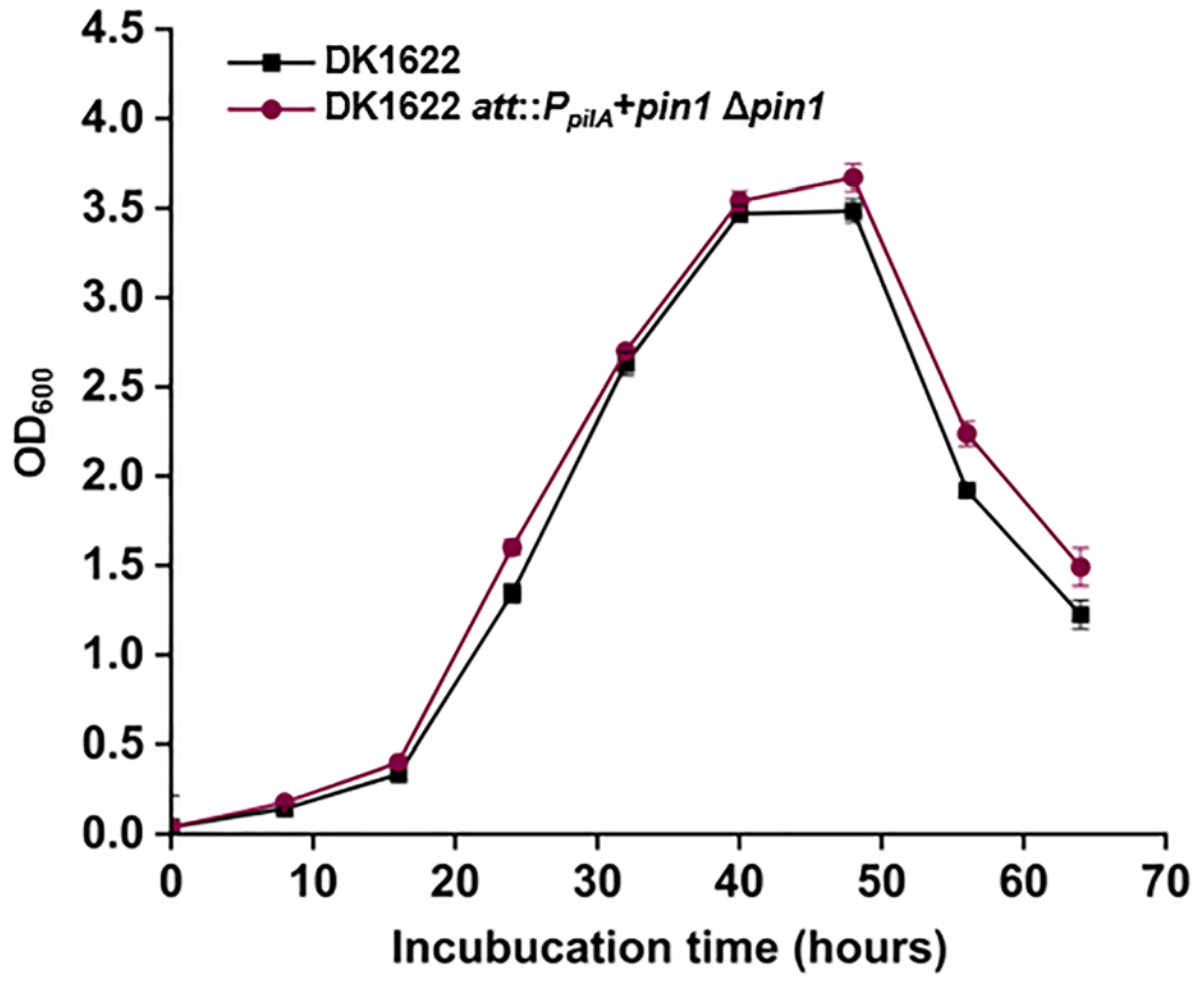

Figure S3. Growth curve of the complementation mutant that complemented by a *pin1* copy inserted at the *attB* site then delete the original *pin1*.

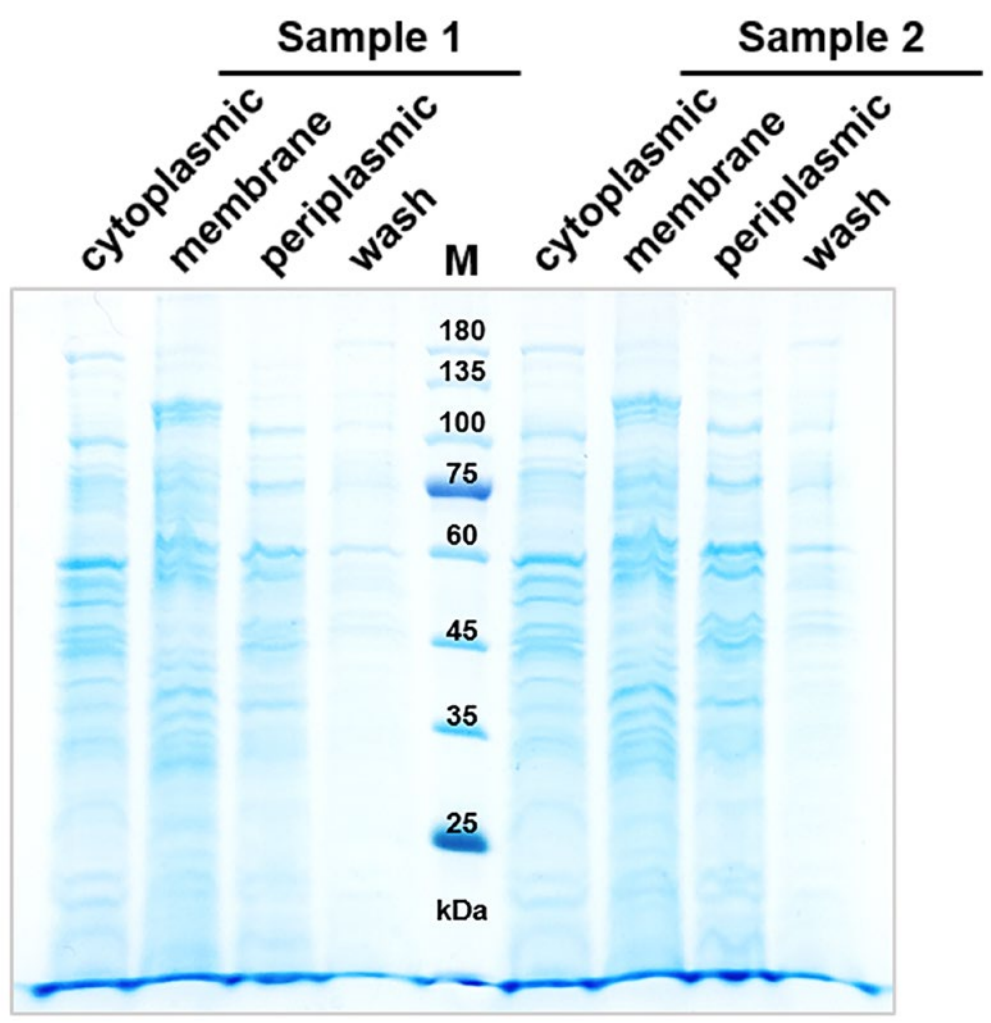

Figure S4. SDS-PAGE of the fractionation results of *M. xanthus* cellular proteins.

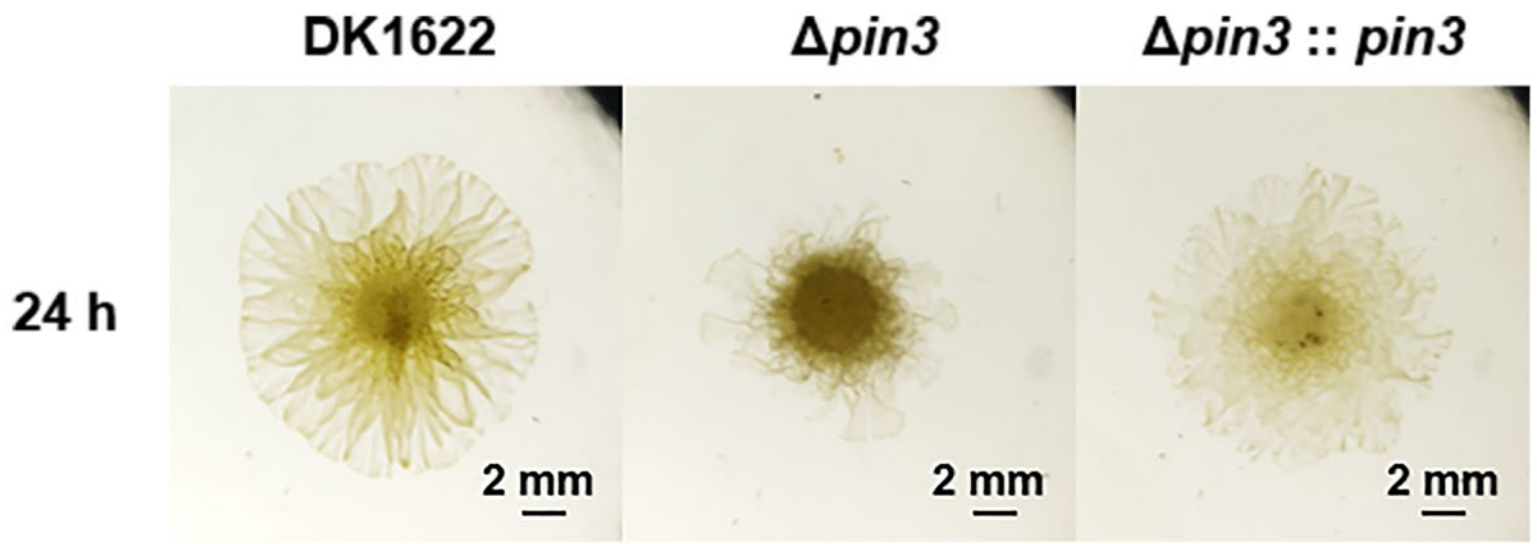

Figure S5. S-motility of *pin3* deletion mutant and its complementation mutant. Expansion of the swarm edge on 0.3% agar CTT plates was observed with a stereoscopic microscope at 24 h. Scale bars: 2 mm.

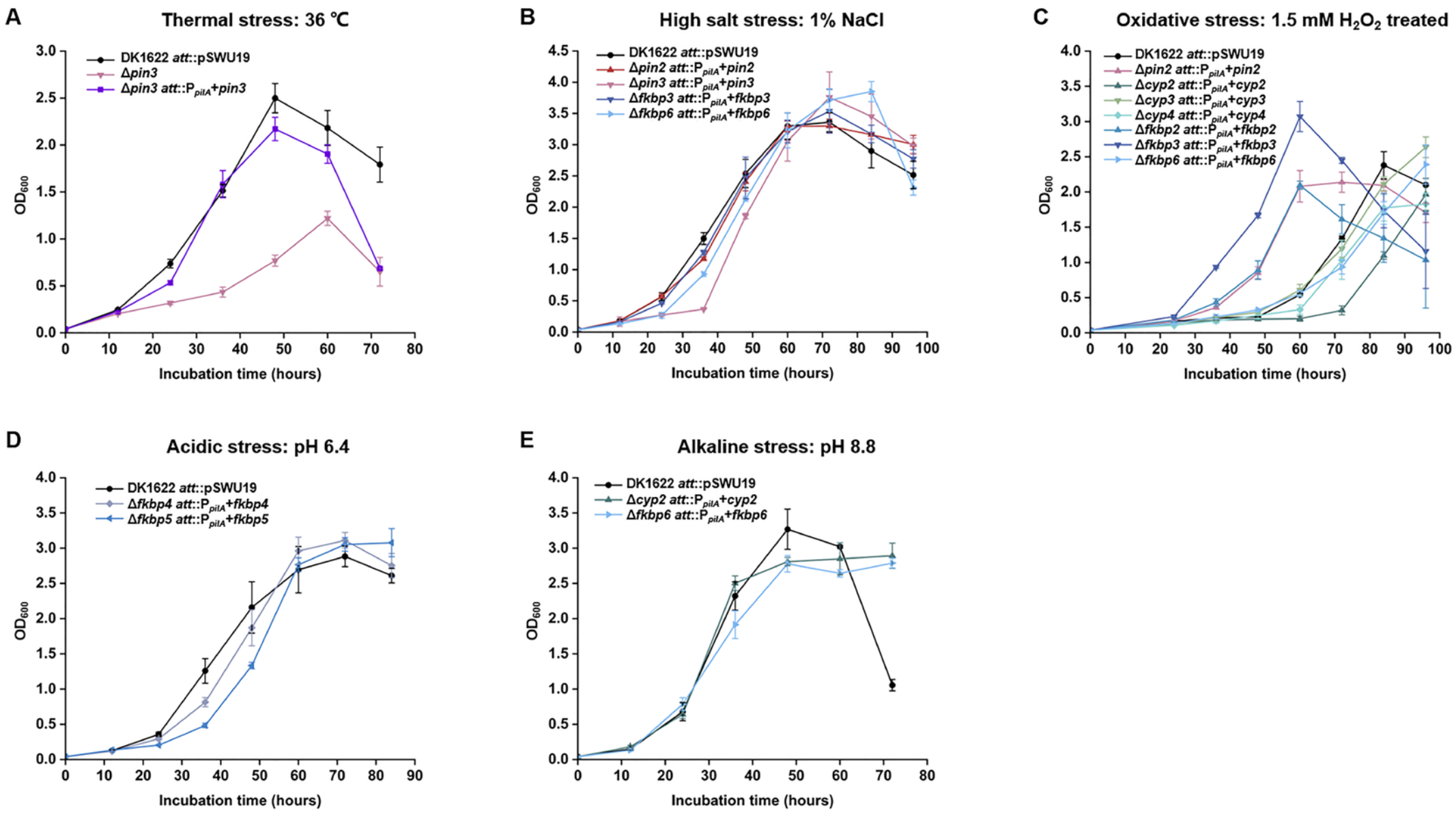

Figure S6. Growth analysis of gene complementation mutants under thermal (A), high salt (B), hydrogen peroxide treatment (C), acidic (D), and alkaline (E) conditions. The complementation mutants were generated by integrating the target gene, driven by the *pilA* promoter, into the *attB* site of the *M. xanthus* genome using plasmid pSWU19, with the corresponding PPIase deletion mutants as the parental strains.

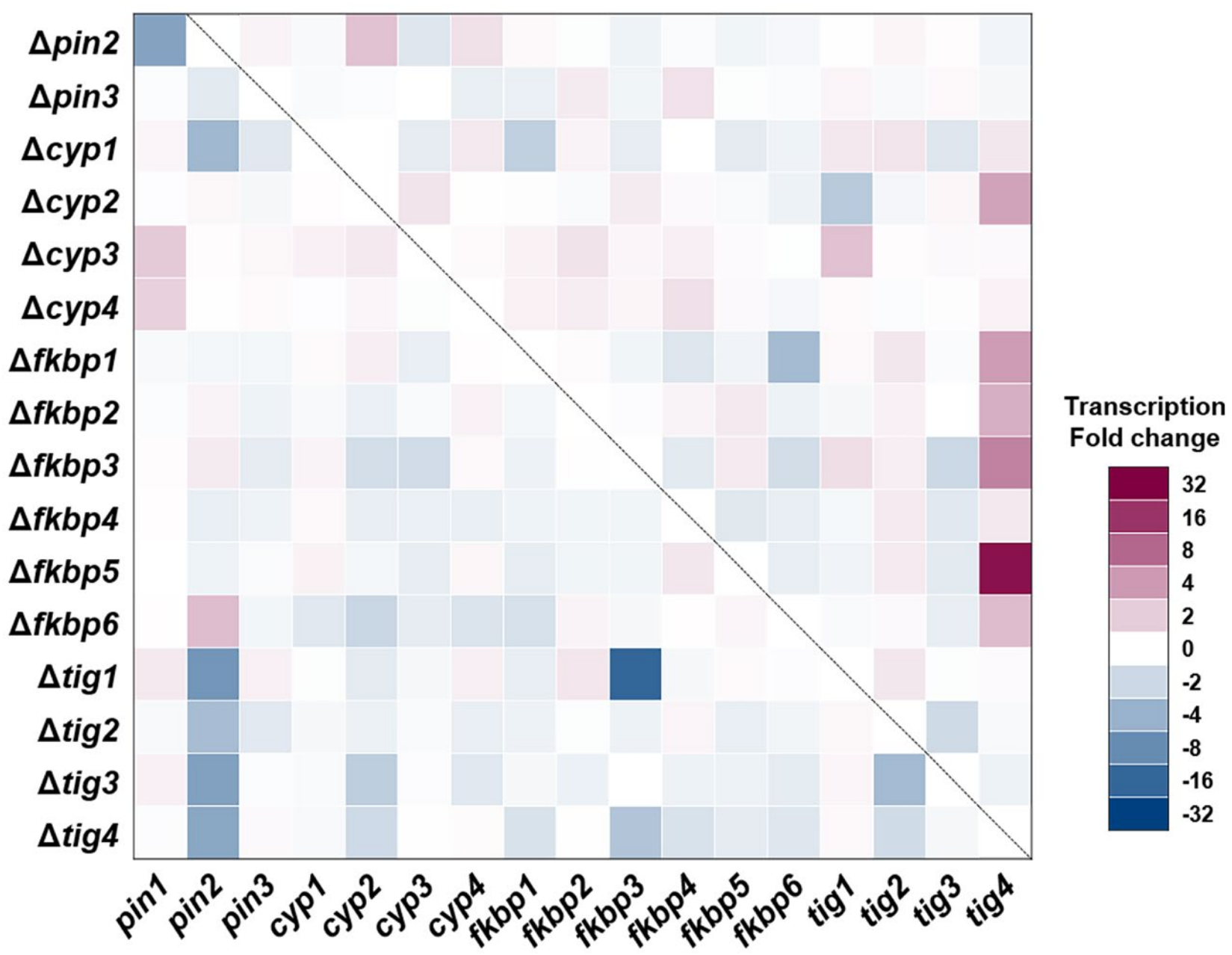

Figure S7. Transcriptional fold changes of PPIase-encoding genes in various deletion mutants at 24 h relative to DK1622. Transcriptional levels of each gene in DK1622 were set as the reference and normalized to 1. Up-regulation is represented in red, and down-regulation in blue.

Supplementary Tables

Table S1. Annotation information of 17 PPIases in *M. xanthus* DK1622.

Number	Locus_tag	Old_locus_tag	Gene ID	Begin	End	Orientation	Protein accession	Protein name	Protein length
Pin1	MXAN_RS02290	MXAN_0468	41357961	531505	532473	minus	WP_026114071.1	peptidylprolyl isomerase	322
Pin2	MXAN_RS02295	MXAN_0469	41357962	532538	533536	minus	WP_171452394.1	peptidylprolyl isomerase	332
Pin3	MXAN_RS12790	MXAN_2644	41360020	3086791	3088356	minus	WP_026113966.1	peptidylprolyl isomerase	521
Cyp1	MXAN_RS05665	MXAN_1176	41358626	1369030	1369776	minus	WP_011551296.1	peptidylprolyl isomerase	248
Cyp2	MXAN_RS18260	MXAN_3764	41361096	4481549	4482145	plus	WP_011553777.1	peptidylprolyl isomerase	198
Cyp3	MXAN_RS18255	MXAN_3763	41361095	4480838	4481545	plus	WP_020478133.1	peptidylprolyl isomerase	235
Cyp4	MXAN_RS36925		41364737	5858976	5861144	minus	WP_020477895.1	peptidylprolyl isomerase	722
Fkbp1	MXAN_RS11625	MXAN_2404	41359788	2796465	2796950	plus	WP_011552479.1	FKBP-type peptidyl-prolyl cis-trans isomerase	161
Fkbp2	MXAN_RS18605	MXAN_3831	41361164	4604419	4604901	minus	WP_011553843.1	FKBP-type peptidyl-prolyl cis-trans isomerase	160
Fkbp3	MXAN_RS19860	MXAN_4085	41361414	5032150	5032626	minus	WP_026114233.1	FKBP-type peptidyl-prolyl cis-trans isomerase	158
Fkbp4	MXAN_RS33175	MXAN_6849	41364038	8401064	8401387	minus	WP_011556774.1	FKBP-type peptidyl-prolyl cis-trans isomerase	107
Fkbp5	MXAN_RS17385	MXAN_3584	41360930	4181247	4181987	minus	WP_026113996.1	FKBP-type peptidyl-prolyl cis-trans isomerase	246
Fkbp6	MXAN_RS34420	MXAN_7110	41364284	8680215	8680994	minus	WP_011557029.1	FKBP-type peptidyl-prolyl cis-trans isomerase	259
Tig1	MXAN_RS09760	MXAN_2013	41359423	2340519	2341796	plus	WP_011552097.1	trigger factor	425
Tig2	MXAN_RS29830	MXAN_6153	41363382	7612526	7613476	plus	WP_225909511.1	peptidylprolyl isomerase	316
Tig 3	MXAN_RS05675	MXAN_1178	41358628	1371020	1372117	plus	WP_216608726.1	peptidylprolyl isomerase	365
Tig 4	MXAN_RS08320	MXAN_1713	41359152	2033033	2034127	minus	WP_011551823.1	peptidyl-prolyl cis-trans isomerase	364

Table S2. Protein property analysis of 17 PPIases in *M. xanthus* DK1622.

Numbered list	Gene	Protein	Protein length	Isoelectric point	Isoelectric point of PPD	Signal peptide (Type)	Membrane spaning domain
**Pin1**	MXAN_RS02290	WP_026114071.1	322	**6.35**	**6.28**	1-22 aa (Sec/SPI)	
**Pin2**	MXAN_RS02295	WP_171452394.1	332	6.6	**9.73**	1-25 aa (Sec/SPII)	
**Pin3**	MXAN_RS12790	WP_026113966.1	521	**5.56**	**5.03**		9-26 aa
**Cyp1**	MXAN_RS05665	WP_011551296.1	248	**6.28**	7.40	1-17 aa (Sec/SPII)	
**Cyp2**	MXAN_RS18260	WP_011553777.1	198	7.35	**8.57**		
**Cyp3**	MXAN_RS18255	WP_020478133.1	235	**10.2**	7.44	1-23 aa (Sec/SPI)	
**Cyp4**	MXAN_RS36925	WP_020477895.1	722	7.2	**5.15**	1-22 aa (Sec/SPII)	
**Fkbp1**	MXAN_RS11625	WP_011552479.1	161	**5.99**	**5.39**		
**Fkbp2**	MXAN_RS18605	WP_011553843.1	160	**4.33**	**4.18**		
**Fkbp3**	MXAN_RS19860	WP_026114233.1	158	**4.55**	**9.93**	1-17 aa (Sec/SPII)	
**Fkbp4**	MXAN_RS33175	WP_011556774.1	107	**8.77**	**9.49**		
**Fkbp5**	MXAN_RS17385	WP_026113996.1	246	**9.37**	**9.58**	1-20 aa (Sec/SPI)	
**Fkbp6**	MXAN_RS34420	WP_011557029.1	259	7.01	7.03	1-15 aa (Sec/SPII)	
**Tig1**	MXAN_RS09760	WP_011552097.1	425	**5.07**	**4.56**		
**Tig2**	MXAN_RS29830	WP_225909511.1	316	**4.54**	**4.06**		
**Tig3**	MXAN_RS05675	WP_216608726.1	365	**4.58**	**4.87**		
**Tig4**	MXAN_RS08320	WP_011551823.1	364	**4.91**	**4.42**		

Table S3. Bacterial strains and plasmids used in this study.

**Designation**	**Genotype or description**	**Source**
	M. xanthus strains	
DK1622	Wild type	D. Kaiser, Stanford University
Δ*pilA*	*M. xanthus* DK1622 Δ*MXAN_5783* (DK10410)	D. Kaiser University of Standford
Δ*aglZ*	*M. xanthus* DK1622 Δ*MXAN_2991* (MxH2265)	P. Hartzell University of Idaho
DK1622 *att*::P_{pilA}+*pin1* Δ*pin1*	Supplement a copy of *MXAN_0468* with the *pilA* promoter at the *attB* site of *M. xanthus* DK1622 and then knockout the original *MXAN_0468* on the genome	This study
Δ*pin2*	Delete *MXAN_0469* in *M. xanthus* DK1622	This study
Δ*pin3*	Delete *MXAN_2644* in *M. xanthus* DK1622	This study
Δ*cyp1*	Delete *MXAN_1176* in *M. xanthus* DK1622	This study
Δ*cyp2*	Delete *MXAN_3764* in *M. xanthus* DK1622	This study
Δ*cyp3*	Delete MXAN_3763 in M. xanthus DK1622	This study
Δ*cyp4*	Delete *MXAN_RS36925* in *M. xanthus* DK122	This study
Δ*fkbp1*	Delete *MXAN_2404* in *M. xanthus* DK122	This study
Δ*fkbp2*	Delete *MXAN_3831* in *M. xanthus* DK122	This study
Δ*fkbp3*	Delete *MXAN_4085* in *M. xanthus* DK122	This study
Δ*fkbp4*	Delete *MXAN_6849* in *M. xanthus* DK122	This study
Δ*fkbp5*	Delete *MXAN_3584* in *M. xanthus* DK122	This study
Δ*fkbp6*	Delete *MXAN_7110* in *M. xanthus* DK122	This study
Δ*tig1*	Delete *MXAN_2013* in *M. xanthus* DK122	Laboratory collection
Δ*tig2*	Delete *MXAN_6153* in *M. xanthus* DK122	Laboratory collection
Δ*tig3*	Delete *MXAN_1713* in *M. xanthus* DK122	Laboratory collection
Δ*tig4*	Delete *MXAN_1178* in *M. xanthus* DK122	Laboratory collection
	E. coli strains	
DH5α	*F-φ80 lac ZΔM15 Δ(lacZYA-arg F) U169 endA1 recA1 hsdR17(rk-,mk+) supE44λ- thi -1 gyrA96 relA1 phoA*	Purchase
BL21	*F_ ompT hsdSB(rB_mB_)dcm gal*(DE3)	Purchase
	Plasmids	
pET28a	Expression vector (Kmr) of protein in *E. coli*	Laboratory collection
pBJ113	Gene replacement vector with KG cassette (KmR)	Laboratory collection
pSWU30ap	Site-specific integration vector at Mx8 *attB* integration site (Aprar)	Laboratory collection
pZJY4111	Autonomously replicating plasmid in *M. xanthus*（Kmr）	Laboratory collection

Table S4. List of primers used in this study.

Primer	Sequence (5'-3')	Use
pBJ113-F	GGGGATCCTCTAGAGTCGACCT	PCR amplification of pBJ13 fragment for construction of gene deletion vectors
pBJ113-R	GGGTACCGAGCTCGAATTCA	
pin1-UF	TGAATTCGAGCTCGGTACCCTCGAAGTCCAGCGCCTTG	PCR amplification the upstream homologous arm of *pin1*
pin1-UR	TACGGAAAGGATGGCGGGACCGCCGTGGGCCTTCCGC	
pin1-DF	GGTCCCGCCATCCTTCCGTA	PCR amplification the downstream homologous arm of *pin1*
pin1-DR	GTcgactctagaggatccccATCCACCGGATGCTGCTGC	
pin2-UF	tgaattcgagctcggtacccAACGCGGCCTTCTCGAAG	PCR amplification the upstream homologous arm of *pin2*
pin2-UR	cgcgcCGACGAAATCGAAGACCGG	
pin2-DF	tcttcgatttcgtcgGCGCGGGGCCTCGTGAGG	PCR amplification the downstream homologous arm of *pin2*
pin2-DR	gtcgactctagaggatccccTGACCAGGCCGAGCATGA	
pin3-UF	tgaattcgagctcggtacccAGTACCCTCGGCCCGGCC	PCR amplification the upstream homologous arm of *pin3*
pin3-UR	ttgccccagaagtgacgacaGTCCCGGCGTCCAACCGT	
pin3-DF	TGTCGTCACTTCTGGGGCA	PCR amplification the downstream homologous arm of *pin3*
pin3-DR	gtcgactctagaggatccccCTCCCGTTCCCGCCGCCG	
cyp1-UF	tgaattcgagctcggtacccACGTCCGCGGTGATGTCG	PCR amplification the upstream homologous arm of *cyp1*
cyp1-UR	taaggtccgcgcgccCGATGGAACGCGCCCTGC	
cyp1-DF	catcgGGCGCGCGGACCTTAGCC	PCR amplification the downstream homologous arm of *cyp1*
cyp1-DR	gtcgactctagaggatccccTGACGCGACGCACCTTCC	
cyp2-UF	tgaattcgagctcggtacccCGCTGGAGAAGCTGCGGG	PCR amplification the upstream homologous arm of *cyp2*
cyp2-UR	atcggacaggGCTACCCTCCCGTGTGGG	
cyp2-DF	ggagggtagcCCTGTCCGATCGCCGCCC	PCR amplification the downstream homologous arm of *cyp2*
cyp2-DR	gtcgactctagaggatccccCTCGCGCTCGGATTCGTC	
cyp3-UF	tgaattcgagctcggtacccGCGGACAAGATGAGCCCG	PCR amplification the downstream homologous arm of *cyp3*
cyp3-UR	acgaacccggtcatgtgGTCGGCGCGGCACCTTAC	
cyp3DF	gacCACATGACCGGGTTCGTCC	PCR amplification the upstream homologous arm of *cyp3*
cyp3-DR	gtcgactctagaggatccccTGACGACGCCGAGGATGC	
cyp4-UF	tgaattcgagctcggtacccCAGCGACCTGACGAACAGCG	PCR amplification the downstream homologous arm of *cyp4*
cyp4-UR	attgacctaggcccgcgccAGCGTCGATTCAGGCGCC	
cyp4-DF	tGGCGCGGGCCTAGGTCAA	PCR amplification the upstream homologous arm of *cyp4*
cyp4-DR	gtcgactctagaggatccccCGACGCACCAGATGAGGC	
fkbp1-UF	gctcggtacccggggatcctGTGGCGCTCACGCCGAGC	PCR amplification the downstream homologous arm of *fkbp1*
fkbp1-UR	agcctacgGCGGGGCTCCTTGCCGAT	
fkbp1-DF	ctcaacgCGTAGGCTCCTGACGCGG	PCR amplification the upstream homologous arm of *fkbp1*
fkbp1-DR	tgcctgcaggtcgactctagTGGCCAGCCACGTGAAGC	
fkbp2-UF	tgaattcgagctcggtacccGCGGATGTTCGACGGCGA	PCR amplification the downstream homologous arm of *fkbp2*
fkbp2-UR	tatgagggccccTTCGGACGGGCGCCTTCG	
fkbp2-DF	cgtccgaaGGGGCCCTCATAGCGCAC	PCR amplification the upstream homologous arm of *fkbp2*
fkbp2-DR	gtcgactctagaggatccccCCACCTGGACCTGCTCCAC	

fkbp3-UF	tgaattcgagctcggtacccGTGGTAGCCATTGGCCTCC	PCR amplification the downstream homologous arm of *fkbp3*
fkbp3-UR	acccagcCGGAGGCGAGGAACTCCG	
fkbp3-DF	ttcctcgcctccgGCTGGGTCAGCCTGCTCC	PCR amplification the upstream homologous arm of *fkbp3*
fkbp3-DR	gtcgactctagaggatccccCAACTGGAAGGAGGCCGC	
fkbp4-UF	tgaattcgagctcggtacccGATGCGCCGTCTCCAACC	PCR amplification the downstream homologous arm of *fkbp4*
fkbp4-UR	gttggAAGCGAGGACCTGATGGCG	
fkbp4-DF	atcaggtcctcgcttCCAACGCTCCTCTGACCGC	PCR amplification the upstream homologous arm of *fkbp4*
fkbp4-DR	gtcgactctagaggatccccTCGGAGCCTGTCTACCCGG	
fkbp5-UF	tgaattcgagctcggtacccGAGGCGTTCGCGAAGGAC	PCR amplification the downstream homologous arm of *fkbp5*
fkbp5-UR	atgaaaggtggatgcGTCAGCGACTCAGGGCGG	
fkbp5-DF	ctgacGCATCCACCTTTCATGAGAACC	PCR amplification the upstream homologous arm of *fkbp5*
fKBP5-DR	gtcgactctagaggatccccCGCAGAACCTGCCCTTCC	
fkbp6-UF	tgaattcgagctcggtacccACGGGTCCTTCGTGCCGA	PCR amplification the downstream homologous arm of *fkbp6*
fkbp6-UR	gcaacgacTCACCGTCTTCACGCAGGA	
fkbp6-DF	tgaagacggtgaGTCGTTGCCCTTCCTTGCTT	PCR amplification the upstream homologous arm of *fkbp6*
fkbp6-DR	gtcgactctagaggatccccGAACGCGGACGGACTTCAC	
pSWU30-U	ggcactggccgtcgttttac	PCR amplification of pSWU30 fragment for construction of expression vectors
pSWU30-D	gtcatagctgtttcctgtgt	
30-pilA-U	aaaacgacggccagtgccCTGGCGAACTACTTCCTGTCCA	PCR amplification the *PilA* promoter in pSWU30
30-pilA-D	CCGCCACCAGCTTCTTCATGGGGGTCCTCAGAGAAGGTTG	
30-A-pin-1-U	CAACCTTCTCTGAGGACCCCCATGAAGAAGCTGGTGGCGG	PCR amplification the *pin1* fragment for ligation with pSWU30
30-A-Pin-1-U	cacaggaaacagctatgacCTACAGCTTCACGTCGACGTT	
q-Pin1-U-2	CCCTCATCGGCGAGAAGCT	Transcription detection of *pin1*
q-Pin1-D-2	AGCAGGCGGTCACGCAGGAT	
q-Pin2-U-3	ACTTCGAACAGGAGCTGTGG	Transcription detection of *pin2*
q-Pin2-D-3	GATAGTCCCCGGACAGCCGCA	
q-Pin3-U-2	AGAAGATGATGGACGTGGTGCG	Transcription detection of *pin3*
q-Pin3-D-2	AAGCGGTTGGCCTCGAAGTA	
q-Cyp1-U-3	GTGGAGGGCAAGCCGCTGTA	Transcription detection of *cyp1*
q-Cyp1-D-3	GAACTGGCTGCCGTTGGTG	
q-Cyp2-U-2	CTGTATGACGGCACGGTGTTC	Transcription detection of *cyp2*
q-Cyp2-D-2	GGCGTGGAGGTGGTGATGAA	
q-Cyp3-U-1	GAAGACGGGTGAGAAGACGC	Transcription detection of *cyp3*
q-Cyp3-D-1	AACTGGCTGCCGTTGGTG	
q-Cyp4-U-3	GCGAGGATGAAGTGGTGGCG	Transcription detection of *cyp4*
q-Cyp4-D-3	TTCACGCAGGCGGGGTTCG	
q-FKBP1-U1	ATGAGGGTCGCCAAGGATTC	Transcription detection of *fkbp1*
q-FKBP1-D1	TGGGTCGTGCTCGCCGTA	
q-FKBP2-U1	GACGAGGGCTACGGCGACTA	Transcription detection of *fkbp2*
q-FKBP2-D1	TCGAAGTGCAGCGTCTTGC	
q-FKBP3-U2	CTGCATAACGAATGCGTTCCC	Transcription detection of *fkbp3*

q-FKBP3-D2	AGCAGCGAGACAGCCAGGAG	
q-FKBP4-U3	TCGATAGCAGCCGGGACC	Transcription detection of *fkbp4*
q-FKBP4-D3	AACAGCAGGGTGGAGTTGG	
q-FKBP5-U1	CGCTGTCGCCCGAGGAGTTGAA	Transcription detection of *fkbp5*
q-FKBP5-D1	CCGCCTGGACCTGCTTGTAG	
q-FKBP6-U3	AGGCGGAGGCTGAGAAGGA	Transcription detection of *fkbp6*
q-FKBP6-D3	ACGCTTGTGCGAGCTGTCG	

Supplementary data

Supplementary data S1. MS-based identification of proteins in different fractions of *M. xanthus* DK1622. The distribution of marker proteins suggests that the extraction of periplasmic fractions was suboptimal, likely compromised by cell lysis. In contrast, the preparation of crude membrane fractions exhibited higher reliability. Since Sec/SPII-type secreted proteins possess a lipid moiety and are mostly anchored in the outer membrane oriented toward the periplasm, it is plausible that these periplasmic proteins are also enriched in the crude membrane fraction. This enrichment may thus serve as a valuable criterion for inferring protein subcellular localization.
